# Management of upper airway edema caused by hereditary angioedema

**DOI:** 10.1186/1710-1492-6-19

**Published:** 2010-07-28

**Authors:** Henriette Farkas

**Affiliations:** 1 3rd Department of Internal Medicine, Faculty of Medicine, Semmelweis University, H-1125 Budapest, Kútvölgyi út 4, Hungary

## Abstract

Hereditary angioedema is a rare disorder with a genetic background involving mutations in the genes encoding C1-INH and of factor XII. Its etiology is unknown in a proportion of cases. Recurrent edema formation may involve the subcutis and the submucosa - the latter can produce obstruction in the upper airways and thereby lead to life-threatening asphyxia. This is the reason for the high, 30-to 50-per-cent mortality of undiagnosed or improperly managed cases. Airway obstruction can be prevented through early diagnosis, meaningful patient information, timely recognition of initial symptoms, state-of-the-art emergency therapy, and close monitoring of the patient. Prophylaxis can substantially mitigate the risk of upper airway edema and also improve the patients' quality of life. Notwithstanding the foregoing, any form of upper airway edema should be regarded as a potentially life-threatening condition. None of the currently available prophylactic modalities is capable of preventing UAE with absolute certainty.

## Introduction

Hereditary angioedema (HAE) is a rare disorder of autosomal dominant inheritance. Its genetic background involves mutation of the gene encoding the C1-inhibitor (C1-INH) or the factor XII protein (FXII) [1,2]. Diverse mutations in the C1-INH gene may result in either inadequately low C1-INH protein (HAE-C1-INH-Type I) or a dysfunctional protein in normal or even excessive amounts (HAE-C1-INH-Type II)[3,4]. This impairment of C1-INH function leads to the activation of all several plasma enzyme cascade (complement, fibrinolytic, coagulation, and kinin) systems controlled by the C1-INH protein and leading to the release of bradykinin. Bradykinin, a vasoactive mediator, enhances capillary permeability and results in the extravasation of plasma into the interstitial space causing edema formation [5]. The diagnosis of HAE is established by family history and clinical manifestation, as well as by determining the antigenic concentration and functional activity of the C4 complement component and C1-INH [3]. A new form (HAE-FXII-Type III) of HAE, described in 2000, occurs predominantly in females and it is not associated with C1-INH deficiency [6]. Mutation of the gene of factor XII can be identified as the underlying cause (HAE-FXII-Type III) in a proportion of cases, but the hereditary background has not been elucidated yet in the remainder (HAE-unknown). The diagnosis is suggested by the family history, the time of the onset of symptoms (after puberty, during pregnancy or the use of oral contraceptives), clinical manifestations, triggering factors (estrogen contraceptives, pregnancy, estrogen hormone replacement therapy), the predominance of females among patients, and the favorable response to progesterone replacement [7]. HAE-Type III is a relatively new entity and accordingly, relevant clinical experience is limited. Therefore, the following information pertains to HAE-C1-INH-Types I and II predominantly, whereas observations related to HAE-Type III of the disease are commented on in the appropriate sections.

All three types of HAE are characterized by recurrent attacks of angioedematous episodes, the severity and frequency of which are highly variable among affected patients. Angioedema can involve both the subcutis and the submucosa. Subcutaneous edema appears on the extremities, face, torso, neck, and genitals and untreated usually resolves spontaneously within several days. Submucosal edema in the gastrointestinal tract may elicit colicky abdominal pain, nausea, vomiting, dysphagia, and diarrhea. These symptoms may mimic an 'acute abdominal catastrophe' and accordingly, afflicted patients may be subjected to unnecessary surgical exploration during attacks [3]. Edema of the airway mucosa may cause life-threatening asphyxia from obstruction. It involves primarily the mucosa of the upper airways and only rarely manifests as pulmonary edema. The exact pathomechanism of this phenomenon is as yet unknown [8-10].

### Edema of airway the upper (UAE) in HAE

#### 1. UAE Mortality

Upper airway edema (UAE) may lead to asphyxia by causing airway obstruction with 30-to 50-per-cent mortality of undiagnosed or inappropriately managed cases [3,11-13]. Bork recorded 29 deaths from suffocation in his series of patients [14]. Before diagnosis, asphyxia tentatively attributed to HAE-C1-INH occurred in six of the 49 families followed up at the Hungarian HAE Center. One family lost three of its members to upper airway obstruction. Although the mortality of UAE has improved dramatically, fatal cases still occur. According to our Hungarian records of the last 15 years, one patient has died of asphyxia due to airway obstruction. Fatal outcome was related to delays in initiating emergency therapy, lack of material and personal preconditions for establishing airway patency, and poor compliance of the patient.

#### 2. UAE Nomenclature

In publications, UAE is referred to simply as 'laryngeal edema'. This term, however, does not describe the condition accurately, as edema often involves the mucosa of the meso-and hypopharynx in addition. Intriguingly, edema-formation spares the mucosa of the nasal cavity and of the paranasal sinuses. The exact anatomical location of the edematous swelling remains uncharted in a large proportion of cases, because patients are only rarely seen by ENT specialists during the attacks. We know of no objective endoscopic evidence on the percentage of true cases of laryngeal edema among the instances labeled as 'laryngeal edema' or on the distribution of these cases according to involvement of the individual anatomical segments of the larynx. In the majority of publications, laryngeal involvement is inferred from indirect signs only (deepening of the patient's voice, hoarseness, aphonia, etc.). Therefore, replacing the term 'laryngeal edema' with 'upper airway edema (UAE)' seems more appropriate and accurate.

#### 3. The diagnosis of UAE

##### 3.1. Clinical manifestations and localization of UAE

Recognizing airway involvement is of primary importance especially for the patients since outcome of an attack is often determined by the promptness of obtaining medical help and receiving early appropriate therapy. The following are potential, subjective symptoms of UAE (ranked in increasing severity):

• Sore, scratchy, itchy throat

• 'Something has stuck in the throat'

• Lump sensation in the throat

• Feeling of throat tightness

• Dysphagia

• Voice changes

• High-pitched or hoarse voice

• Roughness of voice

• Resonant, 'barky' cough

• Stridor

• Dyspnea

• Fear of suffocation

• Aphonia

• Inability to breathe, speak or cough - the patient may grasp his/her throat with thumb and index fingers (i.e. exhibiting the universal choking sign)

• Anxiety and agitation

Edema of the face and lips usually comprises approximately 3% (1.8% in our series) of the episodes of subcutaneous edema [15]. It should be regarded as an important "initial" symptom, because UAE is preceded by facial/labial edema in 15 to 30 per cent of cases. Edematous swelling of the face is more common in HAE-FXII [7]. Edema of the tongue occurs as an isolated phenomenon in about 12% of episodes; however, it may also accompany pharyngeal edema or UAE [16]. The incidence of lingual edema was substantially lower being only 0.02% in our patient population. Remarkably, involvement of the tongue is much more frequent in (similarly bradykinin-mediated) angioedema induced by ACEIs, ARBs and HAE-FXII [17]. UAE may also accompany edema of the extremities, but it only seldom occurs during an abdominal edematous attack [16].

##### 3.2. The clinical course of an UAE attack

In general, UAE evolves into a severe condition over several hours (median: 8.3 hours). Occasionally, the aggravation of pre-existing, mild symptoms takes only minutes owing to the rapid propagation of edema. Alternatively, the episode may follow an inherently fulminant course or its symptoms may resolve spontaneously [15]. The consequences of obstruction become apparent sooner in children because of smaller airway diameter, reduced physiological reserve, and easy fatigability of respiratory muscles. While in adults, edematous swelling of 1 mm thickness causes a 27-per-cent reduction of airway cross-sectional area, it represents reduction of 44% in children and 75% in neonates. Thus, only minor swelling of the airway mucosa can cause severe breathing difficulty in children. Tonsils or adenoid hypertrophy may further aggravate dysphagia and dyspnea [18,19].

##### 3.3. The onset and frequency of UAE symptoms

Although angioedema episodes may occur at any age, their initial onset in HAE-Type I and II is usually observed between 6 and 8 years of age, as well as during adolescence [3]. In HAE-Type III-FXII most symptoms start in the second decade of life [7]. In general, UAE first occurs after the age of 11 and up to 21 years. However, it has been observed as early age as 3 years and as advanced age as 78 years [15]. Our observations confirm this wide age range of occurrence. UAE is uncommon as an initial symptom.. In our series, UAE was the initial manifestation of the disease in 7 out of 132 patients (all are young females). This corresponds to 5.3% of total attack number, which is similar to the 6.3-per-cent proportion seen after diagnosis (n = 489/7044). BORK has described laryngeal edema as the initial symptom in a single, 9-year-old pediatric case only, and the proportion of UAE compared to total attack number was 0.8% (n = 1/125) [15]. The proportions of patients who have ever experienced an UAE are stated by various authors as follows: AGOSTONI-48% [9]; PRUET-50% [20]; CICARDI-78% [12]; BYGUM-55% [21]; BORK-49.6% [15]. We found 56% in our series. UAE occurs more frequently between 21 and 40 years of age, than during either childhood or during adulthood.

##### 3.4. Physical examination

Signs and symptoms of UAE may include:

• Voice changes

• Hoarseness

• Roughness of voice

• Resonant, 'barky' cough

• Stridor

• Dyspnea

• Aphonia

• Anxiety and agitation

• Desperate attempts to breathe, accompanied by intercostal and supraclavicular retractions

• Rapidly progressive cyanosis

• Diminishing respiratory effort

• Loss of consciousness

• Elevated blood pressure and tachycardia, followed by hypotension and bradycardia

• Cardiac arrest

• Death is inevitable if the asphyxiating obstruction is not relieved within 2-5 minutes after its onset

Edema of the soft palate, pharyngeal arch, uvula, and the tongue is easy to ascertain using a spatula, whereas evaluating the condition of the larynx requires endoscopic inspection by a specialist. The latter, however, is uncommon or unfeasible in everyday practice, because the emergency management of UAE is only seldom undertaken by an ENT department. Indirect laryngoscopy, a more straightforward method for the evaluation of the larynx, is difficult to perform in pediatric patients owing to the lack of co-operation. Examination may fail also in adults if the pharyngeal reflex is hyperactive. Furthermore, mechanical contact between the pharyngeal wall and the laryngoscope may result in the progression of edema. Performing indirect laryngoscopy is also difficult in the presence of marked lingual edema. Flexible naso-pharyngo-laryngoscopy may prove appropriate for inspection for airway edema. The visual appearance of the edematous swelling does not differ from that caused by edema of other etiologies (such as inflammatory, allergic)

##### 3.5. Triggering factors

It has been reported that approximately 58% of patients can identify one or more provoking factors of their edematous attacks [9] but this proportion was 85% in our series. Known triggering factors of HAE attacks include mechanical trauma, emotional stress, surgical or diagnostic procedures performed in the head and neck region, physiological fluctuations of sexual hormones (during puberty, menstruation, pregnancy), certain foodstuffs and medicinal products (such as estrogen-containing oral contraceptives and OCs, ACE inhibitors). In UAE, the range of identifiable triggering factors is different [15], the most common being surgical or diagnostic procedures in the head and neck region (such as endotracheal intubation). Dental surgery is a leading cause, potentially associated with fatal UAE [15,22-24]. Before the diagnosis of HAE is established, facial edema or UAE associated with a dental procedure may be mistaken for an allergic reaction to the local anesthetic and this may delay recognizing the true nature of the emergency. Although only rarely emphasized as a potential provoking factor, acute upper airway infection was identified as the triggering factor of UAE in 38% of our 139 patients. Consecutive edema of the face or lips, occurring through indirect mechanisms after mechanical trauma to the head, face or neck often progresses to involve the mucosa of the upper airways. In HAE-Type III-FXII, the introduction of oral contraception and the first pregnancy both can induce the onset of initial symptoms [7].

##### 3.6. Differential diagnosis

The following disorders may be considered if symptoms suggestive of upper airway obstruction ensue in a patient with known HAE: infections (laryngitis, tonsillitis, peritonsillary absecess, epiglottitis), allergic conditions, neoplasm, foreign body, poisoning, autoimmune disease, endocrine disease, gastro-esophageal reflux, and functional abnormalities (CNS depression, neuromuscular dysfunction, peripheral nervous system abnormalities) [11]. These conditions may be distinguished from each other through evaluation of the patient's history, the findings of the physical examination, observed clinical manifestations, and laboratory abnormalities.

Useful clues to clarify the etiology of UAE episodes occurring before HAE is diagnosed include a positive family history, recurrent subcutaneous edema or several-days-long episodes of colicky abdominal pain, accompanied by nausea and vomiting. Another sign of potential differential diagnostic significance is that the drugs conventionally used to relieve airway edema (such as glucocorticoids, antihistamines, and epinephrine) - known to achieve particularly rapid improvement in children in comparison to adults - tend to be ineffective in reducing edematous swelling caused by HAE [25].

#### 4. Management

##### 4.1 Patient education

Informing the patients about UAE is an important component of their education. It must be stressed that virtually any type of airway edema can lead to a potentially life-threatening condition. The manifestations of UAE should be explained in detail, because early recognition of relevant signs and symptoms may affect the outcome of the episode. Care should be taken though not to scare the patient while emphasizing the dangers of UAE. Patients should know that while it is not possible to predict the time of onset and localization of the attack, the risk of developing UAE is increased by edema of the face, lips and tongue of the neck. Additional risk factors include procedures performed in the head and neck region, intratracheal narcosis (in patients with a history of UAE), and age between 11 and 45 years of age [15]. Patients should receive verbal and written information on their disease and its potential manifestations. They should be given a medical information card for emergencies and a patient diary for recording the occurrence of symptoms and the use of treatments. Medication to relieve acute edematous attacks should also be provided (such as C1-INH) - regardless of whether or not the patient has already experienced a severe episode previously.

##### 4.2 Management of UAE event

Whenever it is suspected, the therapy of UAE should be started as early after recognition of onset of initial symptoms as possible. Edema of the face, lips, and neck require immediate intervention. Current options for pharmacotherapy in this treatment setting include plasma-derived C1-INH replacement therapy (pdC1-INH), icatibant (a bradykinin receptor antagonist), and ecallantide (a kallikrein inhibitor). More than 30 years of clinical experience exists using pdC1-INH. Intravenous administration of this agent in a 500 to 1000 U dose is followed by substantial improvement of clinical symptoms within 30 to 60 minutes. In the vast majority of cases, treatment with pdC1-INH usually eliminates symptoms completely within 12 hours [9,12,15,26-29]. Double blind, placebo-controlled studies conducted recently with pdC1-INH concentrate established its recommended dose at 20 U/kg. PdC1-INH concentrate is safe and effective with minimal side effects.. The viral safety of preparations manufactured using innovative pasteurization and nanofiltration technologies is excellent [30]. Treatment with pdC1-INH concentrate does not lead to the formation of antibodies [31] and is safe for children, pregnant women, and nursing mothers [28]. Only limited data are available on its use in HAE-Type III-XII but appears effective in the majority of patients [7]. In our opinion, the best approach is to dispense pdC1-INH concentrate directly to the patients so it is constantly available at home for use on demand. All patients followed up at the Hungarian Center have been provided with pdC1-INH concentrate free of charge. On proposal from the principal of the Center, family practitioners and specialists are authorized to prescribe this medicinal product in the outpatient care setting. Self-administration of emergency medication has substantially improved the patients' quality of life in some countries. Having mastered the technique of intravenous injection, patients may self-administer the drug or have it infused by an appropriate helper. Experience with the self-administration of pdC1-INH concentrate for the treatment of attacks suggests that it is a viable and safe option resulting in faster and more effective treatment of severe angioedema attacks in patients with HAE [32-34].

The effectiveness and safety of the newer agents, icatibant and ecallantide have been demonstrated by clinical studies. Both are to be given by subcutaneous injection, which affords rapid and straightforward administration [35,36]. Experience from long-term follow-up is not yet available, as well as neither of these products has been approved for use in pregnant women, nursing mothers, pediatric patients, nor for self-administration. Notwithstanding this, there is huge demand among patients and doctors alike for additional, safe and effective therapeutic alternatives for HAE attacks and UAE in particular. Although it is not yet available for clinical use, recombinant C1-INH is a new drug for treatment as well [37,38]. If none of the approved medicinal products is available, fresh frozen or solvent-detergent plasma may be used. However, this is no longer considered state-of-the-art therapy and it may even worsen symptoms [39]. Following the successful emergency therapy of UAE, medical observation of the patient is necessary in a facility where intensive care management is available until the complete resolution of symptoms.

If alarming signs of airway obstruction (such as stridor, dyspnea, and signs of respiratory arrest) occur, airway patency should be re-established and oxygen should be administered along with parenteral fluid replacement. Oro-or nasopharyngeal intubation may be useful in unconscious patients. If intubation is not deemed necessary, the patient should be placed in the semi-prone position, head down ('coma') position. If stridor, hoarseness or hypoxaemia are present, immediate intubation is essential,. The extent and localization of the edema may interfere with endotracheal intubation requiring airway patency be restored by surgical intervention.

Cricothyroidotomy is an emergency procedure to prevent death from suffocation caused by upper airway obstruction, when neither endotracheal intubation, nor tracheotomy is feasible. It is relatively easy to perform (the cricothyroid membrane is near to the skin surface) and is only infrequently associated with complications (such as subglottic stenosis, thyroid fracture, haemorrhage and pneumothorax). Commercial cricothyroidotomy sets are available. Inserting a large-bore intravenous catheter through the punctured cricothyroid membrane is a quick, simple, relatively safe and highly effective method. The minimum inner diameter of the tube allowing adequate gas exchange during spontaneous breathing is 3 millimeters.

Percutaneous tracheostomy (PCT) techniques are gaining increasing popularity in surgical ICU wards, especially in 'post-op' rooms or post-anesthesia care units. The indications for PCT are the same as those for standard tracheostomy.

Proper surgical tracheostomy under local anaesthesia may be a prudent approach under controlled conditions. When performing this procedure is not feasible owing to extreme edematous swelling of the neck, cricothyroidectomy is still available for re-establishing airway patency. In 10% of patients, the medical history contains emergency tracheotomy having been performed, occasionally on multiple occasions, before the diagnosis of HAE-C1-INH [9]. Even more astonishingly, fear from the lack of appropriate emergency therapy has prompted some patients to opt for a permanent tracheostomy. In our patient population, previous tracheostomy was identified in the history of 7 of the 132 patients - 2 of them underwent this procedure twice and another 4 on four occasions. In 35 patients with HAE-Type III-FXII 74 episodes of laryngeal edema occured, 3 of these requiring intubation and in 1 case an emergency tracheotomy had to be perfomed [7].

##### 4.3.Prophylaxis

###### 4.3.1 Elimination of triggering factors

Elimination of triggers include the avoidance of mechanical trauma; choosing appropriate sports activities; elimination of mental stress; prevention of infections (sending children too early to nursery schools should be avoided); protective immunization; early recognition of and appropriate symptomatic treatment of infections. The HAE Center should be consulted before starting long-term prophylactic drug therapy. Estrogen-containing oral contraceptives, hormone replacement, ACEIs, and ARBs should be avoided [3].

###### 4.3.2. Short-term prophylaxis

####### 4.3.2.A "Classical" short-term prophylaxis

Considering that surgical and diagnostic (most frequently dental and ENT) procedures performed in the head region may induce UAE, introducing short-term prophylaxis beforehand is warranted [27,29]. The most appropriate and safest approach is to administer pdC1-INH concentrate (500 to 1500 U; 10 to 20 units/kg) one hour before the procedure [27]. According to GOMPELS *et al*, prophylactic use of this drug is recommended within 24 hours before the contemplated intervention [29]. As confirmed by several publications, pdC1-INH concentrate is highly effective for short-term prophylaxis against procedure-related UAE [26-29,40].

Oral treatment is a potential alternative, preferably with attenuated androgens (AAs; danazol, stanozolol, and oxandrolone) or less effectively antifibrinolytics (AFs; epsilon-aminocaproic acid, tranexamic acid), by administering these agents in increased doses for several days before and after the procedure [15,27,29,41]. This option is may be appropriate when the patient is to undergo a minor intervention, when C1-INH concentrate is not available, or if the patient has been receiving the above-mentioned drugs already. Notwithstanding uninterrupted oral dosage with these drugs, however, C1-INH concentrate should be kept in readiness for the duration of the invasive procedure. When pdC1-INH is not available, solvent-detergent treated fresh frozen plasma is a potential alternative (see above under emergency therapy).

####### 4.3.2/B "Alternative" short-term prophylaxis

The follow-up care of patients is primarily focused on the prevention of life-threatening UAE attacks. Alternative prophylaxis - a modified version of short-term prophylaxis - offers gentle and effective means for this purpose. Drug therapy (with AFs, AAs, or pdC1-INH) is administered over the (several-hours-or -days-long) duration of pathological, physiological, or environmental triggering factors (acute airway infection, menses, mechanical trauma or mental stress) - or for several days after the onset of prodromal symptoms. This strategy may prevent the onset of edema or at least mitigate its severity and duration, and prevent UAE [42,43]. Icatibant and ecallantide have not been used for prophylaxis.

###### 4.3.3. Long-term prophylaxis

The objective of long-term prophylaxis (LTP) is to minimize the impact of HAE on everyday life and to prevent the onset of life-threatening attacks. Introducing LTP is justified if the patient's history contains UAEs or the attacks recur frequently. We have observed a positive correlation between attack frequency and the occurrence of UAE and accordingly, the reduction of attack frequency is associated with a lower risk of UAEs. Additional circumstances considered by pertinent guidelines during evaluation of the indications for introducing LTP include availability of emergency therapy, dependence on analgesics, timely access to medical care, number of emergency visits, absenteeism from work or school [27,29,44,45].

Drugs appropriate for LTP include AFs, Aas, and pdC1-INH concentrate [27,29]. The efficacy of AAs is superior to that of AFs but Afs have a better safety profile (although the efficacy evidence supporting their use is less certain) [46]. Nevertheless, AFs are the drugs of choice for the treatment of females and pediatric patients [27,29,42,43,47-50]. AFs are contraindicated in thromboembolic disease and therefore thrombophilia screening is recommended before treatment with this drug is initiated [29]. AAs are more effective and accomplish a statistically significant reduction in the number of edematous episodes [51]. Danazol is most widely used. BORK & BYGUM reported a 92.5-per-cent reduction in the number of laryngeal attacks during the long-term follow up of their 118 patients [51]. It should be noted however, that UAE may still occur despite prophylaxis with these drugs and 5 to 8% of patients do not respond at all to danazol treatment [51,52]. Moreover, the efficacy of danazol may decline over years of use [53]. Treatment may be stared with either escalating or with tapering dose approach [27]. When appropriate, danazol may also be administered to pediatric patients [54]. Undesirable effects can be avoided by administering the lowest effective dose, as well as by monitoring the patient regularly [55-57]. When appropriate, LTP may be implemented using pdC1-INH and this drug should be administered if the oral agents discussed above are ineffective, intolerable, or contraindicated [33,58,59].

Kreuz reported good results with individualized pC1-INH replacement therapy, which prevented UAE and facial edematous attacks [28,30]. Expectedly, recombinant C1-INH concentrate will be another valuable addition to the range of drugs appropriate for LTP. Long-term prophylaxis with progesterone replacement was effective in HAE-Type III-FXII [7].

###### 4.3.4. Intermittent prophylaxis

Under certain circumstances, it may be appropriate to administer prophylaxis for brief periods only. These include the situations where the number and severity of edematous attacks has changed and although the underlying cause for this is suspected, it cannot be eliminated. Additionally, intermittent prophylaxis is recommended during prolonged, critical periods known to provoke edematous attacks (such as starting school, exam periods, outbreaks of infection, changes of the weather during winter months, family problems, puberty, pregnancy, and the like). The drugs used for LTP may be administered as long as the enhanced risk of edematous attacks persists. In pediatric patients, LTP may prove particularly effective and safe for preventing UAE (submitted for publication).

## Summary

Life-threatening complications including UAEs may be prevented and the number of unnecessary abdominal surgeries may be reduced through early diagnosis, patient education, timely recognition of UAE, close observation of the patient, and by ensuring uninterrupted access to emergency therapy. Prophylaxis, regular monitoring, and follow-up all contribute to reducing the incidence of UAE attacks substantially and to improving the safety and quality of life of patients. Notwithstanding the foregoing, any form of upper airway edema should be regarded as a potentially life-threatening condition. Moreover, it is impossible to predict either the time of onset or the dynamics of the attack. Finally, it should be kept in mind that none of the current prophylactic modalities is capable of preventing UAE with certainty.
